# First Fossil Evidence for the Advance of Replacement Teeth Coupled with Life History Evolution along an Anagenetic Mammalian Lineage

**DOI:** 10.1371/journal.pone.0070743

**Published:** 2013-07-25

**Authors:** Xavier Jordana, Nekane Marín-Moratalla, Blanca Moncunill-Solé, Pere Bover, Josep Antoni Alcover, Meike Köhler

**Affiliations:** 1 Institut Català de Paleontologia Miquel Crusafont (ICP), Universitat Autònoma de Barcelona, Barcelona, Catalunya, Spain; 2 Departament de Biodiversitat i Conservació, Institut Mediterrani d'studis Avançats (CSIC-UIB), Esporles, Illes Balears, Spain; 3 Research Associate, Division of Vertebrate Zoology/Mammalogy Department, American Museum of Natural History, New York, New York, United States of America; 4 ICREA (Catalan Institution for Research and Advanced Studies) at Institut Català de Paleontologia Miquel Crusafont (ICP), Universitat Autònoma de Barcelona, Barcelona, Catalunya, Spain; 5 Department of Ecology, Universitat de Barcelona, Barcelona, Catalunya, Spain; Ecole Normale Supérieure de Lyon, France

## Abstract

In mammals that grow up more slowly and live longer, replacement teeth tend to appear earlier in sequence than in fast growing mammals. This trend, known as ‘Schultz's Rule’, is a useful tool for inferring life histories of fossil taxa. Deviations from this rule, however, suggest that in addition to the pace of life history, ecological factors may also drive dental ontogeny. *Myotragus balearicus* is an extinct insular caprine that has been proved to be an excellent test case to correlate morphological traits with life history. Here we show that *Myotragus balearicus* exhibits a slow signature of dental eruption sequence that is in agreement with the exceptionally slow life history of this species, thus conforming to ‘Schultz's Rule’. However, our results also show an acceleration of the absolute pace of development of the permanent incisors in relation to that of the posterior teeth. The rodent-like incisors of *Myotragus balearicus* erupted early not only in relative but also in absolute terms (chronological age), suggesting that feeding characteristics also plays an important role in dental ontogeny. This is in agreement with ecological hypotheses based on primates. Our study documents a decoupling of the pace of development of teeth in mammals that is triggered by different selection pressures on dental ontogeny. Moreover, we show that *Myotragus kopperi* from the early Pleistocene (a direct ancestor of the late Pleistocene-Holocene *M. balearicus*) follows the pattern of first incisor replacement known in living bovids. Hence, the advance in the eruption sequence of the first incisors occurs along the *Myotragus* evolutionary lineage over a period of about 2.5 Myr. To our knowledge, this is the first fossil evidence of an advance of the emergence of the permanent first incisor along an anagenetic mammalian lineage.

## Introduction

Most placental mammals (eutherians) are diphyodonts as they produce two generations of teeth: the deciduous or milk dentition, which erupts around the time of birth, and a permanent dentition, including molars and replacement teeth, that appears after weaning. The replacement pattern is found to conform to the pace of life history over a broad range of taxa [1], a phenomenon coined ‘Schultz's Rule’ In mammals with slower life histories, replacement teeth tend to erupt relatively early and simultaneously with the molars, while in fast growing mammals, molars and replacing teeth erupt sequentially [1]. Humans represent the extreme case of slow dental eruption signature with the second molar erupting only after all the deciduous teeth have been replaced [2]. Schultz [3] proposed that a prolonged growth period places an extra load on deciduous teeth and that species adapt by replacing them relatively early. This rule, hence, states that teeth eruption sequence in eutherians is a functional system adapted to the rate of post-natal growth. Teeth replacement pattern is, thus, useful for making inferences about life histories of fossil taxa [1], [2].

‘Schultz's Rule’ has been proved in primates [4], [5], though exceptions have been found. Studies in lemurs [6] and in tarsius [7] show that their lower anterior teeth erupt relatively and absolutely early regardless of the pace of life history. These studies provide strong support for the foraging independence and the food processing hypotheses [8]. These hypotheses tested across the order Primates suggest an acceleration of the absolute pace of development of the anterior dentition in folivore relative to that of non-folivore primates, facilitating early weaning and independent juvenile foraging. Folivore primates have relatively smaller brains and less complex social interactions, thus survival depends on greater self-sufficiency (foraging independence hypothesis). Moreover, folivorous diet is high in fibrous material and requires extensive processing (food processing hypothesis). Thus, these ecological hypotheses suggest the state of dental development at weaning rather than the pace of life to be a direct target of selection on dental ontogeny [8], [9].

Smith [1] showed that ungulates, as most of primates, replace teeth in a pattern that suits their life history pace. However, generalized herbivores follow ‘Schultz's Rule’ more strictly than specialized herbivores. Hence, the extent to which mammals follow this rule is not clear and ecological factors, mainly diet, have also been claimed to affect dental development.

The Pleistocene bovid *Myotragus balearicus* from Balearic Islands provides an excellent test case for ‘Schultz's Rule’. Recent works have shown the important contribution of this long-living extinct bovid to our understanding of life history evolution [10]–[15]. These studies have shown that certain derived morphological traits of this species, shared by most insular ungulates (‘island syndrome’), such as dwarfism and accentuated hypsodonty, are a by-product of the evolution towards a slow life history that enhances fitness in a resource-limited environment without carnivore predators.

*Myotragus balearicus* presents an extremely modified lower dentition not shared by any other known ruminant that is interpreted as a functional adaptation to increase feeding efficiency under resource limitation [16]. Adults display a single rodent-like evergrowing incisor, a single premolar (p4) and three molars per hemimandible. The second and third incisor, the canine and the third premolar are lost during anagenetic evolution of the *Myotragus* lineage. Their eruptive sequence is also unusual among living bovids, as the permanent incisors and the premolars erupt relatively early [13], [17]. In all living bovids the permanent first incisor appears after the eruption of the second molar and the fourth premolar is the last tooth to emerge after or subsequently to the third molar [18]. However, the permanent incisor in this extinct bovid emerges just after the first molar eruption and the premolar emerges well before the third molar.

*Myotragus balearicus*, hence, appears to conform to ‘Schultz's Rule’. However, Bover and Alcover [17] and subsequently other authors [19] proposed that, like in rodents and lagomorphs, the evergrowing incisor of adult specimens of *M. balearicus* is a primary tooth, specifically the second deciduous incisor, which would explain its early emergence. In a reply, Moyà-Solà et al [20] rebutted this interpretation on the basis of the dental evolution in the *Myotragus* anagenetic lineage and on the standard pattern of dental eruption in extant bovids. They conclude that the evergrowing incisors present in the adult specimens of *M. balearicus* are indeed permanent first incisors that replaced deciduous ones. To explain the relatively early eruption of the permanent first incisors, they suggested an acceleration of the incisor development to cope with resource limitation on islands, in agreement with the abovementioned ecological hypotheses [8]. Here we test whether the pattern (relative order) and pace (absolute age) of dental eruption in *M. balearicus* conforms to ‘Schultz's Rule’ or whether it follows the ecological hypotheses. The slow signature of dental eruption (relative order) in *M. balearicus* may conform to the slow life history of this species. Our recent works using hard tissue histology and demography provided evidence that the *Myotragus* lineage evolved towards a slow life history (slow growth, delayed maturity and long life span) that enhances fitness under density-dependent resource limitation [11]–[15]. Therefore, following the ‘Schultz's Rule’, this shift in the pace of growth of this evolutionary lineage should be accompanied by changes in the sequence of dental eruption. This would provide strong empirical support to ‘Schultz's Rule’. However, an acceleration of the pace of development of the evergrowing incisors of *M. balearicus* leading to an absolute early eruption (chronological age) in the ecological context of chronic resource limitation and high intra-specific competition could provide support toward a feeding adaptation. The present study sheds light on the selection pressures that shape dental ontogeny in mammals.

## Materials and Methods

With the aim of analyzing teeth replacement pattern in the fossil goat-like *M. balearicus* (late Pleistocene to Holocene), an ontogenetic series of mandibles was object of a CT-scan study. The absolute timing of molar development in *M. balearicus* was calculated in a previous work [13] using daily incremental structures of enamel tissue. In the present study, these data were used to calibrate the dental eruption schedule (absolute age) in *M. balearicus*. Moreover, we scanned a juvenile mandible of *M. kopperi* from the early Pleistocene (a direct ancestor of *M. balearicus* in the *Myotragus* anagenetic lineage), as well as a mandible of an extant domestic dwarf goat (*Capra hircus*). CT-scan images were analysed using the software OsiriX v.3.5.1. We used the standard nomenclature for the lower teeth: ‘i’ incisors, ‘p’ premolars and ‘m’ molars, preceded by ‘d’ when referring to a deciduous tooth. We used the original dental homologies for *M. balearicus* proposed by Andrews [21]. The material of *M. balearicus* comes from late Pleistocene-Holocene sites of Mallorca (Cova de Moleta, Cova Estreta and Cova des Moro) and is housed in the collections at the Institut Mediterrani d'Estudis Avançats (IMEDEA), Esporles, Mallorca, and at the Museu Balear de Ciències Naturals (MBCN), Sóller, Mallorca. The mandible of an early Pleistocene species of *M. kopperi* was discovered in 2010 at the Cova des Pas de Vallgornera and is housed at the Institut Mediterrani d'Estudis Avançats (IMEDEA 91270). The mandible of *Capra hircus* comes from the Zoologisches Institut of Kiel (Germany). We obtained permission from these institutions to access the collections. All these specimens were loaned to the authors of this work to carry out radiological study.

## Results

**Figure 1:**
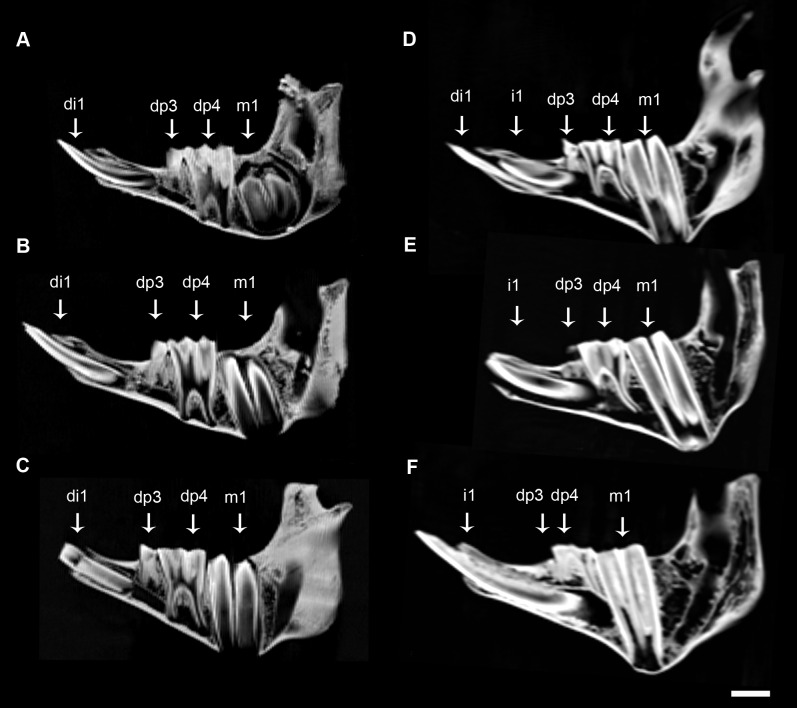
CT-scan images of juvenile lower jaws of *M. balearicus*. Note the development and the replacement pattern of the first incisor. (*A*) MBCN 12592, Cova de Moleta. (*B*) MBCN 12591, Cova de Moleta. (*C*) MBCN 12593, Cova de Moleta. (*D*) IMEDEA 48059, Cova Estreta. (*E*) IMEDEA 85139, Cova des Moro. (*F*) IMEDEA 81013, Cova des Moro. Scale bar  = 1 cm.

The dental eruption schedule of *M. balearicus* mandibles is summarized in table 1. In the youngest specimens of the *M. balearicus* series, two hypsodont incisors of different size, the larger one (di1) located beneath the smaller one (di2), and two deciduous premolars (dp3 and dp4) are already erupted in each hemimandible. The smaller incisor (di2) is likely to fall out very early before m1 eruption. The larger deciduous incisor (di1) shows an open distal base located at the level of mental foramen, before dp3 alveolus. This is the only incisor present in the jaw when m1 begins alveolus emergence (Fig.1A–C) around 6 months.

**Table 1:** Deciduous and permanent teeth present in the hemimandibles of *M. balearicus*.

Age (months)	deciduous teeth	permanent teeth	specimens
0	di1–2^a^, dp3–4	m1 (tooth germ)	MBCN 12591–12592
6	di1, dp3–4	m1 (erupting)	MBCN 12593
9	di1, dp3–4	i1 (tooth germ), m1	IMEDEA 48059
13	dp3–4	i1, m1	IMEDEA 85139–81013
32	dp4	i1, p3^b^–4 (tooth germ), m1–2	IMEDEA 46102–38780
32+		i1, p3^b^–4, m1–2	MBCN 12717
69		i1, p4, m1–3	IMEDEA61221

a The studied specimens lack this tooth most likely by a *postmortem* event. This tooth is present in the similarly aged specimen MNCM 39318 from Bover and Alcover [17].

b Vestigial tooth present in juvenile specimens.

**Figure 2:**
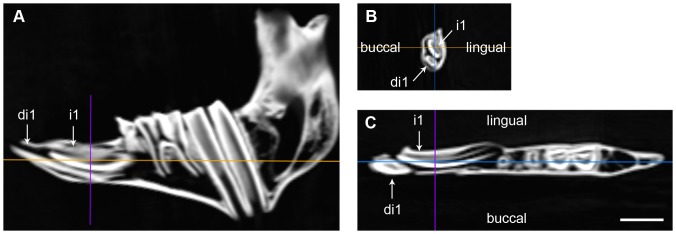
CT-scan images of the mandible IMEDEA 48059 of *M. balearicus*. Replacement of the deciduous first incisor by the permanent one. (*A*) Sagittal plane. (*B*) Coronal plane. (*C*) Transversal plane. Scale bar  = 1 cm.

**Figure 3:**
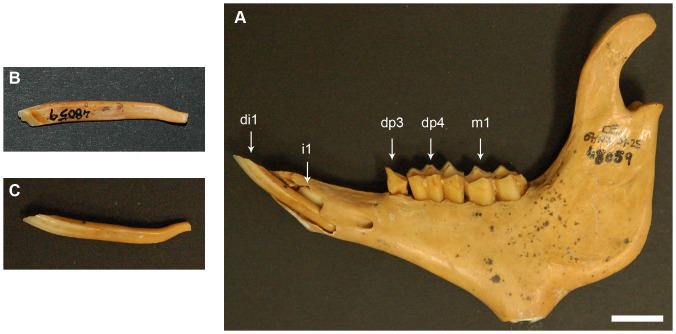
Mandible IMEDEA 48059 of *M. balearicus*. (*A*) Buccal side of the lower jaw showing the germ of the permanent first evergrowing incisor and the erupted deciduous first incisor. (*B*) Lingual side of the deciduous incisor. Note the lack of enamel. (*C*) Buccal side of the deciduous incisor. Scale bar  = 1 cm.

The first molar is fully erupted by 9 months, age at which the adult evergrowing incisor (i1) is already present but not erupted (Fig.1D). This is an elongated incisor (≈23 mm length) located above and at a more lingual position to di1 (Fig.2). The base of i1 is wide and open and is located after mental foramen, just beneath dp3 alveolus, oriented toward the lingual side of the jaw. At this stage, in the specimen IMEDEA 48059 (Fig.3), the incisor already erupted (di1) shows a developed open root that narrows apically.

When m1 crown formation is complete by around 13 months, di1 is already lost. The sole incisors that remain in the jaw (i1) are now erupted (Fig.1E–F). This tooth is greatly enlarged and extends distally beneath dp4 in a more lingual position. During ontogeny, this evergrowing tooth can extend even more distally beneath m1.

**Figure 4:**
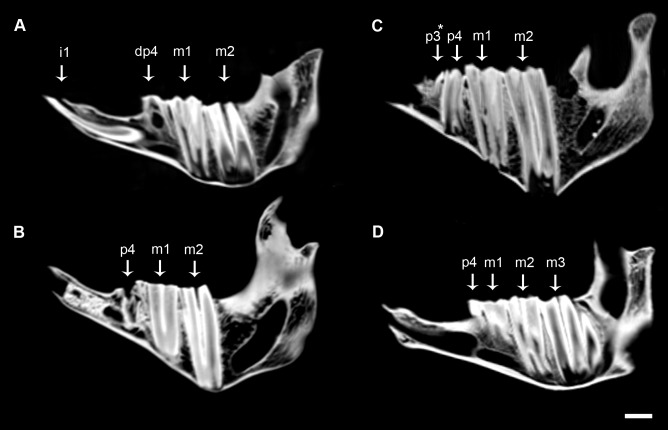
CT-scan images of lower jaws of *M. balearicus*. Note the development and replacement pattern of the fourth premolar. (*A*) IMEDEA 46102, Cova Estreta. (*B*) IMEDEA 38780, Cova des Moro. (*C*) MBCN 12717, Cova de Moleta. * Very reduced conical shaped tooth present in juvenile specimens. (*D*) IMEDEA 61221, Cova des Moro. Scale bar  = 1 cm.

The second molar is fully erupted by about 32 months, and p4 formation starts subsequently (Fig.4A–B). At this stage dp3 is almost depleted and subsequently it is shed. The fourth deciduous premolar falls out and p4 emerges when m2 crown formation is complete. The third deciduous premolar (dp3) is replaced by a very reduced conical shaped permanent premolar (p3) that is lost during adulthood. This vestigial tooth is the result of the evolutionary trend towards the reduction of the premolar series in the *Myotragus* lineage [20]. There is no evidence of m3 formation at this stage (Fig.4C). Finally, the third molar erupts by the age of almost 6 years when the other teeth are already moderately worn (Fig.4D).

**Figure 5:**
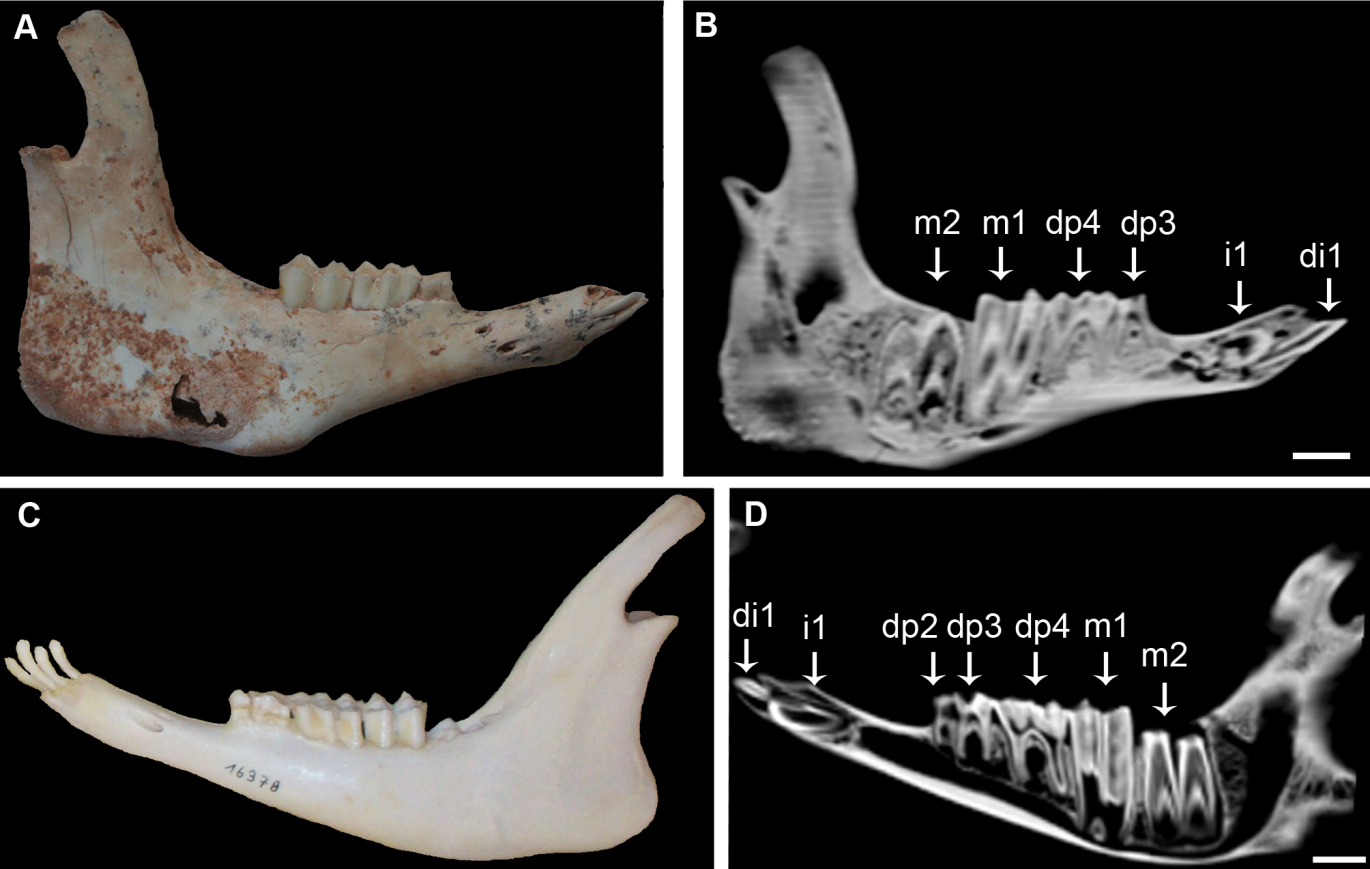
Similarity between the tooth replacement pattern of the early *Myotragus* species and the living goat. Note that in both species the first permanent incisor erupts just after the second molar. (*A*) Mandible of an early Pleistocene *M. Kopperi*, IMEDEA 91270, Cova des Pas de Vallgornera, Mallorca. (*B*) CT-scan image of IMEDEA 91270. (*C*) Mandible of a dwarf *Capra hircus*, Zoologisches Institut, Kiel, Germany. (*D*) CT-scan image of the lower jaw of the living goat. Scale bar  = 1 cm.

As regards the juvenile hemimandible of the early Pleistocene species of *M. kopperi* (Fig.5A, B), it shows the deciduous premolars (dp3 and dp4), the first molar and two deciduous incisors (di1 and di2) erupted and worn, the alveolus of the third deciduous incisor (lost *postmortem*), the germs of the second molar (about to emerge) and the permanent first incisor within the jaw. Therefore, in the early species of the *Myotragus* evolutionary lineage the first permanent incisor (i1) emerged right after the second molar (m2). The same applies in living bovids (Fig.5C, D), but differs from the late Pleistocene-Holocene species (*M. balearicus*), in which the first permanent incisor appears earlier in sequence after the m1 and long before the formation of the m2 begins (see specimen IMEDEA 48059 in figure 2).

## Discussion

Contrary to the suggestion in Bover and Alcover [17], the present study provides further evidence for the successional or secondary nature of the adult evergrowing incisors of *M. balearicus*. Based on the CT-scan study on dental development in *M. balearicus*, the more lingual position within the jaw of the adult incisor germ (i1) relative to the erupted incisor (di1) lends support for its successional nature. The differentiation of a secondary tooth at the lingual margin of the deciduous enamel organ is a constant trait in eutherian dental development [22]. The secondary incisor, hence, always emerges at a slightly more lingual position than the primary one.

However, the strongest evidence for the secondary nature of this evergrowing tooth comes from the fossil record of the *Myotragus* anagenetic lineage. The juvenile mandible of *M. kopperi* from the early Pleistocene clearly shows that there is a replacement of the deciduous incisors, at least in earlier species of this genus.

An important argument for the primary or deciduous nature of the incisors of adult specimens of *M. balearicus* [17] came from embryological studies in rodents and lagomorphs that show that their large evergrowing incisors are deciduous [23]. However, incisor enlargement in mammals can occur at different dental positions and generations [22]. The enlarged lower incisors of rodents and lagomorphs are considered di2 based on the development of small, abnormal di1 on the prenatal jaws, which are probably subsequently resorbed [22]. Nevertheless, this is very different from what happens in *M. balearicus*, in which di1 is a functional highly hypsodont incisor that falls out by around the first year of age. Moreover, this tooth is noticeably wider and longer relative to earlier species of the genus *Myotragus* [20].

The extant artiodactyl vicuña (*Vicugna vicugna*) shows parallel evolution to *M. balearicus* in the incisor enlargement [24]. The vicuña displays three deciduous elongated incisors with open root bases and without enamel on the lingual side of the crown, which are similar to di1 of *M. balearicus*, though not as elongated. Three permanent evergrowing incisors with morphology very similar to that of the adult incisor of *M. balearicus* replace the primary teeth of vicuña.

Based on all these arguments, the adult incisor of *M. balearicus* should be considered a successional tooth, specifically i1. Therefore, it can be established that replacement teeth (incisors and premolars) in the fossil bovid appear relatively earlier in sequence than in living bovids. In addition, the advance in the eruption sequence of the i1 occurs along the anagenetic lineage of the genus *Myotragus* over a period of about 2.5 Myr. The first permanent incisor in the early Pleistocene *M. kopperi* mandible emerged right after the second molar, as in living bovids. However, in the late Pleistocene-Holocene species (*M. balearicus*) the replacement of deciduous incisors by the first permanent incisor has advanced in the sequence, just after the first molar eruption. This is, to our knowledge, the first evidence from the fossil record of advance of incisor replacement along an evolutionary mammalian lineage.

According to the study of Smith [1] on patterns of tooth eruption sequence in mammals, *M. balearicus* exhibits a slow signature typical of species with an age of m1 emergence (a proxy for rate of postnatal growth) by around one year and a median life span of about 30 years. These data conform to the slow life history described for the extinct species. The present study shows that m1 of *M. balearicus* is fully erupted at 9 months and crown formation is complete around 13 months. Moreover, the potential life span for *M. balearicus* has recently been estimated as around 27 years using tooth cementum annuli [15] and 34 years using bone histology [14]. Both, the values of m1 emergence and life span are unusual among extant bovids. They are even rare among artiodactyls in general, except for some larger, slower-growing species such as hippopotamus (*Hippopotamus amphibius*) that show the same slow sequence signature of tooth eruption as does *M. balearicus* as a consequence of an atypically delayed juvenile period in the context of a slow life history.

As regards the pace of dental eruption (absolute age), the large evergrowing incisor (i1) of *M. balearicus* erupted by an age of around one year, which is the standard time of eruption in similar-sized continental bovids, or even slightly earlier [18], [25]. This incisor grew relatively fast as it took at most three months to attain its important length before eruption. Therefore, the adult incisor did not only grow fast in relative terms but also in absolute terms. However, the posterior teeth (molars and premolar) of *M. balearicus* exhibit a slow pace of development that doubles the eruption time of similar-sized wild mainland caprines [13]. Thus, the molars of *M. balearicus* need more than twice the time to reach the same crown height of i1 just before eruption. This slow pace of development of posterior teeth in *M. balearicus* is coupled with the prolonged growth period and delayed age at maturity within the framework of a slow life history [11]–[13]. This decoupling between the pace of anterior and posterior tooth development suggests that the state of dental development at weaning might also be target of selection in agreement with ecological hypotheses [8]. The absolute early eruption of the large rodent-like evergrowing incisor of *M. balearicus* is likely to be selectively advantageous under the special ecological conditions on islands, as suggested by Moyà-Solà et al [20]. In an ecological context of high intraspecific competition for scarce resources, selection favours increased harvesting efficiency as shows the highly specialized dentition of adults [12]. Our results indicate that selection also operated at an early age leading to dental precocity at weaning. The early eruption of the adult evergrowing incisors provides the mechanical requirements for processing more abrasive, tougher foodstuff (food processing hypothesis), in addition to facilitate weaning and independent juvenile foraging (foraging independence hypothesis).

Among living ruminants, two deer species, the white-tailed deer (*Odocoileus virginianus*) and the reindeer (*Rangifer tarandus*), also show very early emergence of their first permanent incisors, though not of the premolars [1]. This accelerated incisor development in these two deer species is most likely related to dental precocity in the context of harsh high-latitude environment, rather than to the pace of life.

Many studies have documented decoupling among dental eruption pattern, dental development pace, and life history pace [7], [8], [26]. However, the present study is the first to document a decoupling between the pace of anterior and posterior tooth development.

In summary, this study provides a plausible explanation for the early emergence of the rodent-like evergrowing incisor and the premolar of *M. balearicus*. There is evidence that the pattern of tooth development in this fossil species conforms to ‘Schultz's Rule’. Thus, the relatively early eruption of the replacement teeth in *M. balearicus* is a functional adaptation to a prolonged growth period in the context of insularity. The early replacement of the anterior teeth (incisors) also appears to be dictated, or at least partially, by resource constraints imposed by density-dependent selection. Moreover, we provide first empirical evidence from the fossil record of advance of incisor replacement coupled with a shift towards a slow life history along the evolution of a mammalian lineage.
